# Factors associated with *allergy traits* around the 2nd year of life: a brazilian cohort study

**DOI:** 10.1186/s12887-022-03772-7

**Published:** 2022-12-08

**Authors:** Alessandra Karla Oliveira Amorim Muniz, Cecilia Claudia Costa Ribeiro, Elcio Oliveira Vianna, Hellen Cristina Oliveira Amorim Serra, Joelma Ximenes Prado Teixeira Nascimento, Viviane Cunha Cardoso, Marco Antonio Barbieri, Antonio Augusto Moura da Silva, Heloisa Bettiol

**Affiliations:** 1 Department of Public Health, Federal University of Maranhão – UFMA, São Luís, Maranhão Brazil; 2 Ribeirão Preto Medical School, University of São Paulo – USP, Ribeirão Preto, São Paulo Brazil

**Keywords:** Allergic rhinitis, Atopic dermatitis, Food allergy, Pregnancy, Birth, Structural equation modeling

## Abstract

**Background:**

Allergic status has been strongly influenced by early exposures; however, allergic diseases are hard to measure in early life. Thus, this study proposed a latent variable allergy traits around the second year of life and analyzed pre- and perinatal factors associated with this phenomenon.

**Methods:**

The study used data from the BRISA birth cohort, Ribeirão Preto, Brazil (*n* = 3644). The theoretical model included: family allergy (history of allergic rhinitis, atopic dermatitis, and asthma); gestational period variables (socioeconomic status, mother’s skin color, pregestational body mass index – BMI, smoking, gestational diabetes, and hypertension); birth variables (gestational age, 5-minute Apgar score, birth weight, type of delivery), and early life factor (exclusive breast feeding). The outcome was allergy traits around the 2nd year of life, a latent variable deduced from the shared variance among medical diagnosis of allergic rhinitis, atopic dermatitis, and food allergy. The model was analyzed by structural equation modeling.

**Results:**

Higher socioeconomic status (SC = 0.256; *p* < 0.001) and higher family allergy values (SC = 1.224; *p* < 0.001) were associated with higher allergy trait values. Hypertension during pregnancy was associated with higher values (SC = 0.170; *p* = 0.022) and exclusive breast feeding (SC = -0.192; *p* < 0.001) with low allergy trait values.

**Conclusion:**

Although socioeconomic and environmental factors were associated with allergy traits around the 2nd year of life, the family component of allergy was the exposure that best explained this outcome.

**Supplementary Information:**

The online version contains supplementary material available at 10.1186/s12887-022-03772-7.

## Background

Atopic dermatitis, food allergy and allergic rhinitis are the most prevalent allergic diseases in the world [1]. The prevalence, complexity and severity of these diseases are increasing, especially among children and young adults. The World Health Organization estimates that approximately 200 to 250 million people worldwide have food allergies and 400 million have allergic rhinitis [2].

These allergic diseases are chronic and usually begin in early childhood, with a process known as atopic march [1]. Atopic march is the natural history of allergic manifestations, characterized by a typical sequence of clinical signs and atopic diseases, beginning with atopic dermatitis (AD) and food allergy, followed by allergic asthma and rhinitis [3, 4].

Exposures during conception, pregnancy and the first years of life are determinants for the development of the immune system and are associated with a higher risk of allergic sensitization and diseases [5]. These exposures include smoking during pregnancy and prematurity [6], birthweight [5], maternal obesity [7], cesarean delivery, Apgar score [8], no exclusive breast-feeding [9], living in an industrialized city, and atopy of parents [10].

Despite recognizing pre-birth factors as being associated with allergic diseases, most epidemiological studies have been conducted on school-aged children, with a reduced number of studies on preschoolers [11, 12]. An association of cesarean delivery, low 5-minute Apgar scores, large for gestational age and moderate prematurity with allergic rhinitis [8] and food allergy [13] has been observed among Swedish children. In these studies, the first medical diagnosis of food allergy occurred at about 1.6 years of age [13] and the diagnosis of allergic rhinitis at 5.7 years [8].

Recognizing that allergies in early childhood are a condition of difficult diagnosis, we proposed a latent variable to minimize the measurement error and to better approach the allergic diseases co-existing in preschool children. Thus, this study tested the latent variable *allergy traits* around the 2nd year of life and analyzed pre- and perinatal factors associated with it.

## Methods

### Study design

This study was part of a prospective cohort entitled “Etiological factors of preterm birth and consequences of perinatal factors on the child’s health: birth cohorts in two Brazilian cities, Ribeirão Preto and São Luís - BRISA” [14] carried out in Brazil. In the present study, we used data from Ribeirão Preto at two time points: at the birth of children in 2010 (baseline) and follow-up at the second year of life [14]. The city of Ribeirão Preto has a Municipal Human Development Index of 0.800 and is located in an industrialized region of Brazil (southeast) [15].

### Population and sample

**Fig. 1:**
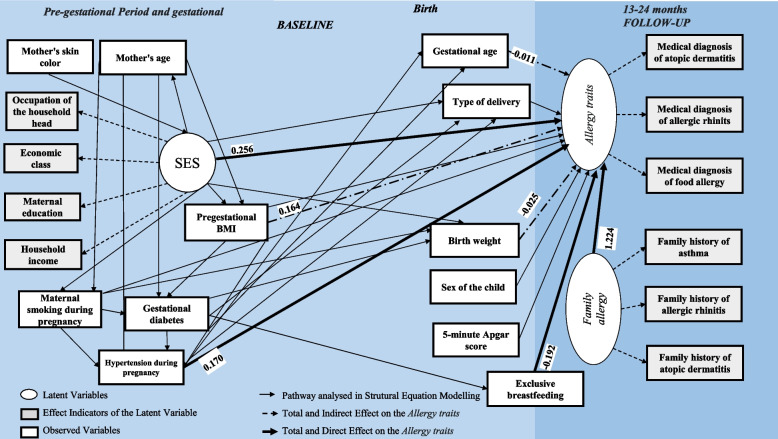
Proposed theoretical model, Ribeirão Preto-SP, 2010–2013. Mother’s skin color was the most distal variable with an effect on *socioeconomic status* (SES), which is directly associated with the other variables of the model. *Family allergy* directly affects *allergy traits* around the 2nd year of life and is affected by the mother’s age and pregestational body mass index – BMI. Prenatal factors (pregestational BMI, smoking during pregnancy, gestational diabetes and hypertension during pregnancy) are intrinsically related to a positive effect on *allergy traits* [10, 18]. Prenatal factors would affect gestational age, type of delivery, overweight at birth and Apgar score, which would be directly related to *allergy traits* [8, 13]. Exclusive breast feeding would be a protective factor for allergic diseases

The Ribeirão Preto birth cohort included all births at public and private hospitals in the city in 2010 (*n* = 7794). Follow-up occurred from 13 months of life, between February 2011 and September 2013, comprising 3805 children and their mothers. After the non-inclusion criteria (stillborns, early deaths, and multiple births) and exclusion one (non-respondents to questions about allergy), the final sample consisted of 3644 individuals (Figure 1 - Supplemental Material).

### Data collection procedures

At baseline, health professionals and undergraduates monitored daily the information of medical records in maternity hospitals of Ribeirão Preto and invited postpartum women living in this city for at least 3 months to interview. For the follow-up, all mothers included in 2010 were contacted to participate in a new interview and health assessment of their children.

The questionnaire applied to mothers during the 24-hour postpartum period asked about: maternal age (years), monthly household income (multiples of the Brazilian minimum wage which was approximately U$$ 290,00 in 2010), maternal education (years of schooling), occupation of the household head, and economic class according to the Criterion of Economic Classification Brazil (D/E - poorest, C, and A/B - wealthiest) [16].

Smoking during pregnancy and self-reported diabetes and hypertension based on medical diagnosis were categorized as dichotomous (yes or no). Information about the following variables was collected from the medical records: mother’s age, self-reported skin color, gestational age, type of delivery (vaginal or cesarean section), weight (kg), 5-minute Apgar score, and sex of the child. Gestational age was calculated based on the first day of the last menstrual period or obstetric ultrasound for the children followed since prenatal care when the difference in gestational age was > 10 days between the use of the first day of the last menstrual period versus obstetric ultrasound [17]. Gestational age and birth weight were treated as continuous variables and the 5-minute Apgar scores as categorical: low (< 7) or standard (≥7).

At the second year of life follow-up, the duration of exclusive breast-feeding was obtained using the question: “How long was your child exclusively breastfed? (read to the mother: exclusive breast-feeding is just breast milk, no tea, water, other milk, other beverages or food)”. The algorithm used to calculate the duration of exclusive breast-feeding (days) considered the substitution of this information when the number of days reported in the questions about the introduction of water, milk, and infant formula was lower than the time reported by the mother. Therefore, exclusive breast-feeding was dichotomized as yes (≥180 days) or no (< 180 days).

The variable *family allergy* was obtained from the questionnaire of 2nd year follow-up with questions about family history of asthma (Does your baby have a father, mother, or sibling with asthma?), family history of allergic rhinitis (Does your baby have a father, mother, or sibling with a nose allergy or allergic rhinitis?), and family history of atopic dermatitis (Does your baby have a father, mother or sibling with atopic dermatitis or eczema?).

The investigation of *allergy traits* was based on the questionnaire of the follow-up with questions about a medical diagnosis of atopic dermatitis (Since birth, has any doctor diagnosed atopic dermatitis - eczema; skin allergy characterized by a rash with intense itching, which goes back and forth, in any area of the body except around the eyes and nose, and in the diaper region?), of allergic rhinitis (Has any doctor ever told you that your baby has allergic rhinitis?), and of food allergy (Has any doctor ever told you that your baby has an allergy to any food?).

### Statistical analysis

The variables were categorical and are presented as relative and absolute frequencies using the Stata 15.0 software.

Given follow-up losses, all estimates were weighted. The following procedures were performed: 1) comparison of all variables of this study concerning attendance or not in the follow-up of the cohort by the chi-square test; 2) calculation of the probability of children attending follow-up (1 = yes, 0 = no) in a logistic regression model using as predictor variables those that showed significant differences (maternal age, education and skin color, household income, economic class, hypertension during pregnancy, pregestational BMI, type of delivery, and gestational age), and 3) calculation of the inverse probability of participation in the follow-up, which was used as the weight.

#### The latent variables

*Socioeconomic status* was a latent variable with evidence of validation in a previous study in the BRISA cohort, deduced by the shared variance among the maternal education level, occupation of the household head, household income, and economic class [18].

*Family allergy* was a latent variable deduced from the shared variance among family history of asthma, allergic rhinitis, and atopic dermatitis.

The latent variable *allergy traits* around the 2nd year of life was evaluated as an outcome of interest, deduced from the shared variance among medical diagnosis of allergic rhinitis, diagnosis of atopic dermatitis, and diagnosis of food allergy.

The proposed latent variables were evaluated by confirmatory factor analysis (CFA). Model fit was assessed by the following fit indices: a) *p* > 0.05 and an upper 90,0% confidence intervals < 0.08 for the Root Mean Square Error of Approximation (RMSEA); b) comparative fit index (CFI) and Tucker-Lewis index (TLI) (> 0.95) [19].

#### Structural equation modeling

Figure 1 presents the theoretical model proposed for the analysis of pregnancy and birth factors associated with *allergy traits* based on structural equation modeling (SEM). SEM is a statistical tool that uses CFA and simultaneously estimates a series of regression equations, evaluating the direct and indirect associations among a set of variables with one or more outcomes. The models were classified as good depending on the value of the fit indices described for CFA, using the Mplus® 7.0 software. The modindices command was used [20] to check the need to improve the originally proposed model fit.

### Ethical aspects

The study was approved by the Research Ethics Committee of the University Hospital, Ribeirão Preto Medical School, University of São Paulo, Brazil (protocol number 11157/2008).

## Results

The sociodemographic, lifestyle and reproductive health characteristics of the mothers are shown in Table 1. White women belonging to classes C and A/B, with a household income of 3 to < 5 Brazilian minimum wages predominated in the sample. The sociodemographic and health characteristics of the children are listed in Table 2. There were higher rates of children with a medical diagnosis of allergic rhinitis (22.7%) and atopic dermatitis (8.5%) than food allergy (5.4%) reported by the parents in the 2nd year of life. Rhinitis and asthma were also reported as allergic diseases with higher percentages in the family (Table 2).

**Table 1:** Sociodemographic characteristics, lifestyle and reproductive health of mothers in the BRISA cohort, Brazil, 2010–2013

Variable	Ribeirão Preto	
	N	%
Skin color		
White	2105	57.8
Black	364	10.0
Brown	1125	30.9
Yellow	32	0.9
No information	18	0.5
Years of schooling		
0–4	134	3.7
5–8	709	19.5
9–11	2009	55.1
12 or more	776	21.3
No information	16	0.4
Occupation of the household head		
Unskilled manual	867	23.8
Semiskilled manual	1602	44.0
Skilled manual	193	5.3
Office functions	378	10.8
Higher level professional	327	9.0
Administrator/owner	141	3.9
No information	136	3.7
Household income (minimum wages)		
< 1	8	0.2
1 and < 3	911	25.0
3 and < 5	1125	30.9
≥ 5	1072	29.4
No information	528	14.5
Economic class		
D - E (poorest)	264	7.2
C	1665	45.7
A - B (wealthiest)	1531	42.0
No information	184	5.1
Smoking during pregnancy		
No	3250	89.1
Yes	394	10.8
Hypertension during pregnancy		
No	3176	87.2
Yes	459	12.6
No information	9	0.2
Gestational diabetes		
No	3388	93.0
Yes	248	6.8
No information	8	0.2
Type of delivery		
Vaginal	1470	40.3
Cesarean	2174	59.7
Pregestational BMI		
Low weight	243	6.7
Eutrophy	1653	45.3
Overweight	775	21.3
Obesity	421	11.5
No information	552	15.1
Total	3644	100

Brown: mulatto/cabocla/brunette

Yellow: Asian/Indigenous

**Table 2:** Sociodemographic and health characteristics of children in the BRISA cohort, Brazil, 2010–2013

Variable	Ribeirão Preto	
	Median	25th–75th Percentile
Gestational age (weeks)	38.5	37–40
Birth weight (kg)	3.2	2.9–3.5
	**N**	**%**
Sex		
Male	1818	49.9
Female	1826	50.1
Low 5-minute Apgar score (< 7)		
No	3624	99.4
Yes	20	0.5
Exclusive breast feeding (EBF)		
No	2691	73.8
Yes	947	26.0
No information	6	0.2
Medical diagnosis of allergic rhinitis		
No	2815	77.2
Yes	829	22.7
Medical diagnosis of atopic dermatitis		
No	3334	91.5
Yes	310	8.5
Medical diagnosis of food allergy		
No	3446	94.6
Yes	198	5.4
Family history of allergic rhinitis		
No	1533	42.1
Yes	2090	57.3
No information	21	0.6
Family history of atopic dermatitis		
No	3220	88.4
Yes	403	11.1
No information	21	0.6
Family history of asthma		
No	3114	85.5
Yes	518	14.2
No information	12	0.3

*EBF* Exclusive breast feeding, Breast milk must be the infant’s only intake for the first 6 months of life, without additional tea, water, other milk, other beverages or food

The proposed model had good fit (Table 3). All indicators of the latent *allergy traits* around the 2nd year of life showed significant *p* values and factor loadings close to 0.5, except for the food allergy indicator (SC = 0.225; *p* < 0.01) (Table 4).

**Table 3:** Adjusted model indicators. Ribeirão Preto - SP, 2010–2013

Indicators	Ribeirão Preto Model
Root mean square error of approximation (RMSEA)	0.023
RMSEA (90% CI)	0.021–0.026
*p*-value	1.0
Comparative fit index (CFI)	0.979
Tucker-Lewis index (TLI)	0.968

**Table 4:** Standardized coefficient, standard error, and *p*-value of latent variables. Ribeirão Preto - SP, 2010–2013

Latent variable	Indicator variables	Ribeirão Preto		*p*-value
		SC	SE	
*Socioeconomic status*	Occupation of the household head	0.645	0.011	
	Household income	0.769	0.012	*< 0.001*
	Maternal education	0.765	0.012	
	Economic class	0.846	0.010	
*Family allergy*	Family allergic rhinitis	0.629	0.038	
	Family atopic dermatitis	0.556	0.040	*< 0.001*
	Family asthma	0.295	0.038	
*Allergy traits*	Atopic dermatitis	0.471	0.048	
	Allergic rhinitis	0.470	0.045	*< 0.001*
	Food allergy	0.225	0.052	

*SC* standardized coefficient, *SE* standard error

High *socioeconomic status* (SC = 0.256; *p* < 0.001), *family allergy* (SC = 1.224; *p* < 0.001), and hypertension during pregnancy (SC = 0.170; *p* = 0.022) were associated with higher *allergy trait* values in children. Exclusive breast feeding was associated with lower *allergy trait* values around the 2nd year of life (SC = -0.192; *p* < 0.001) (Table 5). Pregestational BMI, mediated by hypertension during pregnancy, was associated with *allergy traits* around the 2nd year of life (SC = 0.059; *p* = 0.042). Gestational age (SC = -0.011; *p* = 0.004) and birth weight (SC = -0.025; p = 0.004) had an indirect association mediated by exclusive breast feeding, with the lowest *allergy trait* values around the 2nd year of life (indirect association, data not shown in tables).

**Table 5:** Standardized coefficient, standard error, and p-value of total, direct and indirect effects. Ribeirão Preto - SP, 2010–2013

Cohort/ City	*Allergy traits*	Total effect			Indirect effect			Direct effect		
		SC	SE	***p***-value	SC	SE	***p***-value	SC	SE	***p***-value
Ribeirão Preto	***Socioeconomic status***	**0.256**	**0.049**	**< 0.001**	0.444	0.112	**< 0.001**	−0.188	0.118	0.111
	***Family allergy***	**1.224**	**0.127**	**< 0.001**	0.000	0.000	1.000	**1.224**	**0.127**	**< 0.001**
	**Pregestational BMI**	0.090	0.049	0.064	**0.164**	**0.066**	**0.013**	−0.074	0.076	0.327
	Maternal smoking during pregnancy	−0.064	0.076	0.400	0.047	0.071	0.506	−0.111	0.105	0.289
	Gestational diabetes	−0.048	0.085	0.574	0.025	0.017	0.136	−0.072	0.088	0.409
	**Hypertension during pregnancy**	**0.170**	**0.074**	**0.022**	−0.006	0.023	0.798	**0.176**	**0.085**	**0.039**
	Gestational age	0.023	0.045	0.612	0.085	0.063	0.178	−0.062	0.077	0.420
	Birth weight	0.020	0.056	0.719	−0.082	0.052	0.110	0.103	0.075	0.171
	Type of delivery	0.007	0.075	0.928	0.002	0.046	0.970	0.005	0.088	0.954
	5-min Apgar score	−0.287	0.206	0.163	0.000	0.000	1.000	−0.287	0.206	0.163
	**Exclusive breast feeding**	**−0.192**	**0.055**	**< 0.001**	0.000	0.000	1.000	**−0.192**	**0.055**	**< 0.001**

*SC* standardized coefficient, *SE* standard error

## Discussion

The latent variable *allergy traits* around the 2nd year of life was a good indicator of convergent validity to represent the three most common allergic diseases in childhood. Pregnancy and birth factors were associated with *allergy traits*, with higher *socioeconomic status*, *family allergy*, and hypertension during pregnancy being associated with higher values. In contrast, exclusive breast feeding was associated with lower *allergy trait* values around the 2nd year of life.

Ribeirão Preto city had an increase in air pollution derivate from heavy vehicle traffic and the mechanization of significant production of alcohol and sugar [21], which may contribute to the high taxes of allergic rhinitis in our sample.

*Allergy traits* around the 2nd year of life were deduced from the variance shared by atopic dermatitis, rhinitis, and food allergy, and the factorial loading of the indicators of latent variables was greater than 0.4, except for food allergy (0.233), with *p* < 0.001. This result supports the knowledge that atopic allergic diseases already march together in early life.

*Family allergy* was the variable with the strongest association with *allergy traits* around the 2nd year of life, suggesting that genetic factors underlie the developing allergic diseases. International guidelines and consensus have described that rhinitis, eczema, asthma, or food allergy in at least one first-degree relative (mother, father, or sibling) increase the risk of these outcomes in the child [22–24].

Higher *socioeconomic status* values were associated with higher *allergy trait* values around the 2nd year of life, mediated by *family allergy.* Other studies have also identified that better socioeconomic level and a family history of allergies are associated with the development of allergic rhinitis, food allergy, and atopic dermatitis in children [12, 25]. According to the hygiene hypothesis, children from families that have more access to urban and industrialized environments, with better hygiene and lower microbiological exposure that decrease infection rates and immune regulation in childhood are likely to have a higher prevalence of allergic disease [26].

As an evolution of the hygiene hypothesis, the ‘disappearing microbiota’ hypothesizes that some factors related to the development of society, such as treated water, cesarean delivery, and use of infant food formulas, contribute to the imbalance of microorganism types in the gut as a consequence of the development of diseases. Evidence indicates that the initial analysis of the microbiota of children who later develop allergic diseases reveals less diversity compared to children who do not develop such diseases [27, 28]. Unexpectedly, children who were born by cesarean section did not have higher *allergy trait* values around the 2nd year of life*.*

Hypertension during pregnancy was directly associated with higher *allergy trait* values around the 2nd year of life*.* Furthermore, hypertension during pregnancy was a mediator of the association of higher pregestational BMI with *allergy traits* around the 2nd year of life. Thus, the effect of hypertension during pregnancy on *allergy traits* might be explained by increased inflammation in the uterus, resulting in an irregular distribution of T cells, increasing IgE in the fetus and the risk of allergic disease, a mechanism already proposed for preeclampsia [29].

Breast feeding has been demonstrated to protect against allergic diseases by promoting the development of a more diversified microbiota, based on the exchange of microorganisms between the mother and baby and the composition of breast milk itself. The bioactive and immunomodulatory components of breast milk, in addition to reinforcing this healthy colonization in the newborn, can favor the development of an initial defense mechanism that compensates for the immaturity of the immune system [9].

The results of systematic reviews have shown that smoking during pregnancy may increase the risk of allergic rhinits [30], of asthma and of early wheezing [31]. Contrary to expectations, in the present study *s*moking during pregnancy was not associated with *allergy traits around the 2nd year of life. This finding is similar to that of a previous study with pregnant women in the BRISA cohort - São Luís that did not identify an association of maternal smoking with asthma* [18].

The main limitation of the present study was that the diagnosis of allergic diseases was made on a questionnaire, in which the child’s guardian reported allergy traits based on medical diagnosis. Thus, to reduce the measurement error of allergic diseases, we used the latent variable, represented by the correlation between allergic diseases and not by an isolated allergic indicator.

Among the study limitations, BRISA Cohort in 2010 did not have questions on prebiotic/probiotic or Vitamin D use, which would have permitted us to test the association of these components with allergic status.

In addition, people with higher *socioeconomic status*, especially those with higher educational level, may report family allergy more frequently. This group has better access to health services, with the possibility of better detection of the outcome by the doctor at earlier ages. Finally, the factorial load of food allergy indicator variable was considered low. This may reflect a measurement error given its difficult diagnosis, especially among children aged less than 2 years.

## Conclusions

Regarding the strengths of the present study, the diagnosis of allergy as a latent variable deduced from the variance shared among the primary allergic diseases in preschoolers (atopy, food allergy, and allergic rhinitis) represents a methodological difference in relation to other longitudinal studies [8, 13], which, despite evaluating explanatory factors, started from a single allergic indicator (rhinitis, atopy, or food allergy). Thus, longitudinal analysis by structural equations, in addition to enabling the analysis of explanatory factors at different moments (prenatal, birth, and second year of life), reduced the measurement error of this difficult to measure outcome by any of its indicators alone and, consequently, of the associated factors.

Our findings indicate that investigations of risk factors for allergy should begin in the first stages of life and suggest that intensified prenatal care able to diagnose hypertension during pregnancy and the encouragement of exclusive breast feeding are important for allergy prevention, especially in families that already have a family allergy and among high socioeconomic status children.

The association between higher *socioeconomic status* and *allergy traits* around the 2nd year of life suggests the role of the hygiene hypothesis in the allergic march at an early age. Maternal obesity and hypertension during pregnancy represent an inflammatory risk for *allergy traits* around the 2nd year of life, while exclusive breast feeding is associated with a protective immune response. As the primary determinant, a history of *family allergy* was the exposure that best explained the *allergy traits* around the 2nd year of life.

## Supplementary Information

**Additional file 1: Figure S1.** Flow diagram of the BRISA birth cohort, Ribeirão Preto, Brazil.

## Data Availability

The datasets used and/or analyzed during the current study are available from the corresponding author on reasonable request.

## Abbreviations

AD: Atopic Dermatitis
BMI: Body Mass Index
CFA: Confirmatory Factor Analysis
CFI: Comparative Fit Index
EBF: Exclusive breast feeding
RMSEA: Root Mean Square Error of Approximation
SC: Standardized coefficient
SE: Standard error
SEM: Structural Equation Modeling
TLI: Tucker-Lewis Index
