# Supplementary figures and images for: Factors associated with allergy traits around the 2nd year of life: a brazilian cohort study
Source: BMC Pediatr. 2022 Dec 8;22:703. doi: 10.1186/s12887-022-03772-7 (PMC9733343; doi:10.1186/s12887-022-03772-7)

## SUPPLEMENTAL MATERIAL

Figure 1. Flow diagram of the BRISA birth cohort, Ribeirão Preto, Brazil

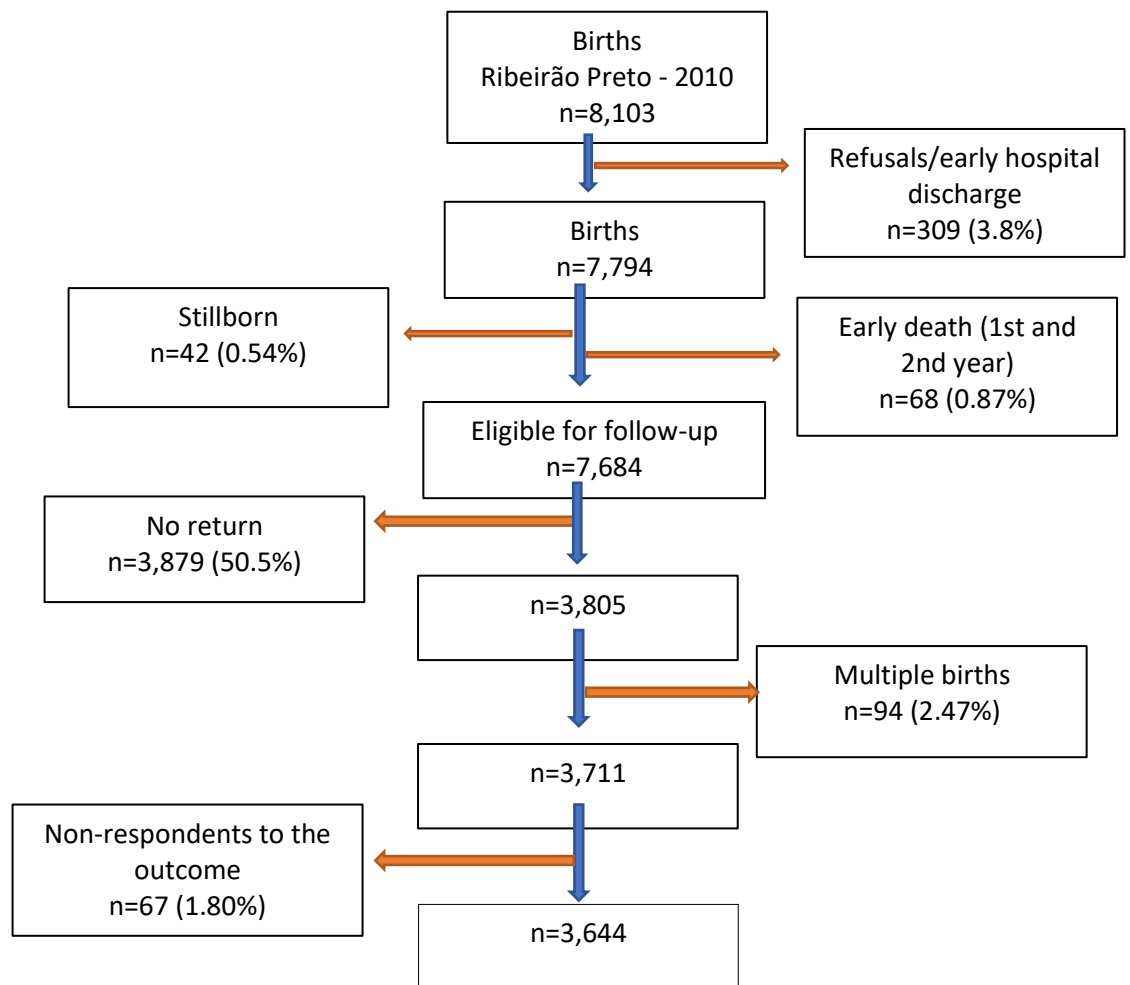

Supplement: Supplementary file 1 — Additional file 1: Figure S1. Flow diagram of the BRISA birth cohort, Ribeirão Preto, Brazil. [file 12887_2022_3772_MOESM1_ESM.pdf]
